# Bacterial diversity of the American sand fly *Lutzomyia intermedia* using high-throughput metagenomic sequencing

**DOI:** 10.1186/s13071-016-1767-z

**Published:** 2016-08-31

**Authors:** Carolina Cunha Monteiro, Luis Eduardo Martinez Villegas, Thais Bonifácio Campolina, Ana Clara Machado Araújo Pires, Jose Carlos Miranda, Paulo Filemon Paolucci Pimenta, Nagila Francinete Costa Secundino

**Affiliations:** 1 Laboratory of Medical Entomology, René Rachou Research Centre (FIOCRUZ-MG), Belo Horizonte, Minas Gerais Brazil; 2 Centro de Pesquisas Gonçalo Moniz (CPqGM)-Fundação Oswaldo Cruz (FIOCRUZ), Salvador, Bahia Brazil

**Keywords:** *Lutzomyia intermedia*, Native microbiota, Metagenomics

## Abstract

**Background:**

Parasites of the genus *Leishmania* cause a broad spectrum of diseases, collectively known as leishmaniasis, in humans worldwide. American cutaneous leishmaniasis is a neglected disease transmitted by sand fly vectors including *Lutzomyia intermedia*, a proven vector. The female sand fly can acquire or deliver *Leishmania* spp. parasites while feeding on a blood meal, which is required for nutrition, egg development and survival. The microbiota composition and abundance varies by food source, life stages and physiological conditions. The sand fly microbiota can affect parasite life-cycle in the vector.

**Methods:**

We performed a metagenomic analysis for microbiota composition and abundance in *Lu. intermedia,* from an endemic area in Brazil. The adult insects were collected using CDC light traps, morphologically identified, carefully sterilized, dissected under a microscope and the females separated into groups according to their physiological condition: (i) absence of blood meal (unfed = UN); (ii) presence of blood meal (blood-fed = BF); and (iii) presence of developed ovaries (gravid = GR). Then, they were processed for metagenomics with Illumina Hiseq Sequencing in order to be sequence analyzed and to obtain the taxonomic profiles of the microbiota.

**Results:**

Bacterial metagenomic analysis revealed differences in microbiota composition based upon the distinct physiological stages of the adult insect. Sequence identification revealed two phyla (Proteobacteria and Actinobacteria), 11 families and 15 genera; 87 % of the bacteria were Gram-negative, while only one family and two genera were identified as Gram-positive. The genera *Ochrobactrum, Bradyrhizobium* and *Pseudomonas* were found across all of the groups.

**Conclusions:**

The metagenomic analysis revealed that the microbiota of the *Lu. intermedia* female sand flies are distinct under specific physiological conditions and consist of 15 bacterial genera. The *Ochrobactrum, Bradyrhizobium* and *Pseudomonas* were the common genera. Our results detailing the constituents of *Lu. intermedia* native microbiota contribute to the knowledge regarding the bacterial community in an important sand fly vector and allow for further studies to better understand how the microbiota interacts with vectors of human parasites and to develop tools for biological control.

## Background

Parasites of the genus *Leishmania* cause a broad spectrum of diseases in humans worldwide, collectively known as leishmaniasis. American cutaneous leishmaniasis (ACL) is a neglected disease found in 18 countries and approximately 60,000 new cases are diagnosed every year [1]. In Brazil, it is present in all states [2] and is prevalent mainly in the northern and northeastern regions. Even though several *Leishmania* spp. have been described, *Leishmania braziliensis* is the main species causative of ACL and can be transmitted by different sand fly species in distinct endemic geographical regions. In fact, there are several sand fly species recognized as proven vectors of *L. braziliensis*, including *Lutzomyia intermedia*, *Lutzomyia whitmani*, *Lutzomyia neivai*, *Lutzomyia migonei*, *Lutzomyia wellcomei* and *Lutzomyia complexa* [3].

In the southeastern region of Brazil, *Lutzomyia intermedia* is the main vector of *L. braziliensis* [4, 5]. This opportunistic species is associated with forests, nature reserves and modified domestic and peridomestic environments, such as animal shelters [6–8].

The female sand fly can acquire or deliver *Leishmania* spp. parasites while feeding on a blood meal, which is required for nutrition, egg development and survival. All events involved in parasite transformation, development, survival and establishment of vector infection occur in the digestive tract. A variety of microorganisms, such as fungi and bacteria, can interact with *Leishmania* spp. in the vector and during their transmission to a vertebrate host. Bacterial communities have been studied to understand how bacteria interact with parasites. The insect microbiota varies by food source and life stage; during the immature stage, sand fly larvae ingest microorganisms from the soil environment. Furthermore, adult sand flies have the opportunity to ingest microorganisms, either from plant sap or sticky honeydew from aphids. Alternatively, adult females also ingest yeasts and bacteria from their vertebrate hosts used as blood source [9–13]. All these microbes will contribute to form their midgut microbiota.

In 2002, Volf et al. [10] studying *P. duboscqi* showed microbiota modulation due to blood meal. Two days after the ingestion of a blood meal, there was an increase in bacterial number in the midgut followed by a decrease after blood meal digestion. This fact is in line with other studies performed with other nematoceran insects (i.e. *Aedes aegypti* and *Anopheles gambiae*) showing an increase in their midgut microbiota after a blood meal [14, 15]. More recently, in vitro effect of microbiota on *Leishmania* spp. survival was demonstrated showing that *Serratia*, *Bacillus* and *Haemophilus parainfluenzae* induced lysis of promastigote forms [12, 16, 17]. Moreover, sand flies pre-fed with *Pseudozyma*, *Asaia* or *Ochrobactrum* showed a reduction in parasite survival [18].

Here, we performed a metagenomic analysis for microbiota composition and abundance in female adult sand flies, specifically in *Lu. intermedia* originated from an endemic area in Brazil. This study establishes basic knowledge for future studies to understand the role of microbiota during the course of *L. braziliensis* infection in this important vector.

## Methods

### Sand flies

*Lutzomyia intermedia* adults were collected using CDC light traps [19] from a locality in Corte de Pedra, State of Bahia, Brazil (longitude 59°30'W; latitude 13°26'S); a hilly region (600 m above sea level) covered by different stages of secondary Atlantic forests. Corte de Pedra is a village with human interference involved with cocoa and clove plantations. The light traps were placed around animal shelter in April 2014.

### Specimen preparation

The collected sand flies were anesthetized at -20 °C for a few seconds and had their surfaces carefully sterilized to prepare for serial passage as follows: 10 s in 70 % ethanol, 1 min in 1 % hypochlorite and 1-min wash in PBS three times [20]. The specimens were morphologically identified according to Young & Duncan [21]. Sand fly midguts were dissected under a stereoscope and separated into groups according to their physiological condition: (i) absence of blood meal (unfed = UN); (ii) presence of blood meal (blood-fed = BF); and (iii) presence of developed ovaries (gravid = GR). The pooled samples (300 of each group) were stored in micro centrifuge tubes with (100 μl) of RNAlater® (Qiagen, Hilden, GER) at 4 ° C for later RNA extraction and metagenomic analysis.

### Metagenomic process

#### RNA extraction

Samples previously stored in RNAlater were washed in distilled water using a 35 μm nylon filter prior to RNA extraction. RNA extraction was performed in two steps. First, the pooled samples were ground in TRI Reagent® RT (Molecular Research Center, Inc., Cincinnati, USA) and chloroform with Teflon pestle and then incubated at room temperature for 15 min. The samples were centrifuged at 15,294 rcf at 4 °C for 15 min followed by the addition of 70 % ethanol to the supernatant. The supernatant was transferred to a column (RNeasy Mini Kit, Qiagen, Hilden, GER) and processed according to the manufacturer’s protocol. RNA quality was assessed using a NanoDrop spectrophotometer (Thermo Fisher Scientific, Massachusetts, USA), and 50 ng/μl of RNA was used to synthetize cDNA using the Reverse Transcription Kit (Qiagen, Hilden, GER). cDNA was diluted 10×, stored at -20 °C and submitted for high-throughput Illumina sequencing.

#### Illumina HiSeq sequencing

cDNA from the three sand fly experimental groups were sent to the DNA Service Platform (University of Illinois, USA), where the samples were sequenced (http://www.biotech.illinois.edu/htdna/services-equipment). The 16S ribosomal cDNA primers used were the following: CS1_27F sense: 5'-ACA CTG ACG ACA TGG TTC TAC AAG AGT TTG ATC CTG GCT CAG CS2_534R and antisense: 5'-TAC GGT AGC AGA GAC TTG GTC TAT TAC CGC GGC TGC TGG-3' (http://hmpdacc.org).

#### Sequence analysis and taxonomic profiling

The reads corresponding to the targeted 16S rRNA V1-V3 region were QC processed using the tools implemented within the Quantitative Insights Into Microbial Ecology (QIIME) software v1.7.0 pipeline [22]. Briefly, primer sequences were removed, and reads were trimmed at the extremities using a Phred value Q20. High quality reads (≥ 250 bp) were further processed to remove chimera sequences using ChimeraSlayer. Sequences within an identity threshold of 97 % were clustered into Operational Taxonomic Units (OTUs) using the UCLUST [23] pipeline. The most abundant sequence within each of the clusters was selected as representative, and its taxonomic affiliation was assigned using the Ribosomal Database Project (RDP) classifier [24] via QIIME considering a minimum confidence value of 80 %.

The bacterial community composition of each experimental group was compiled considering only OTUs encompassing at least a 0.5 % relative abundance. The cut-off value was set to address rare sequences that most likely corresponded to random sequencing errors (eg, previously reported thresholds for community composition tables in other insect models vary from 1 to 0.04 % [25, 26].

## Results

### High-throughput Illumina sequencing

Sequencing revealed a total of 587,144 valid reads, which were grouped as follows: UN: 117,492; GR: 44,318; and BF 425,334. After the sequences were quality checked and assembled, the UN group was clustered into 66 OTUs, the GR group into 30 OTUs, and the BF group into 40 OTUs; only 12, 9 and 8 OTUs in the UN, GR and BF groups respectively, were considered valid bacterial OTUs according to the cut-off value. An average of 2 % reads were unassigned and 2.9, 1.4, and 1.4 % of OTUs fell below the cut-off line in the UN and GR and BF groups, respectively.

### Diversity of bacterial microbiota

Bacterial metagenomic analysis revealed differences in microbiota composition based upon the distinct physiological stages of the adult insect. Sequence identification revealed two phyla (Proteobacteria and Actinobacteria), 11 families and 15 genera; 87 % of the bacteria were Gram-negative, while only one family and two genera were identified as Gram-positive (Table 1).

**Table 1:** Taxonomic classification of the metagenomic OTUs of *Lutzomyia intermedia*

Family	Genus (Gram stain)
*Bradyrhizobiaceae*	*Bradyrhizobium* (−)
*Brucellaceae*	*Ochrobactrum* (−)
*Comamonadaceae*	*Pelomonas* (−)
*Enterobacteriaceae*	*Enterobacter* (−)
	*Erwinia* (−)
	*Kluyvera* (−)
	*Serratia* (−)
	Unidentified genus
*Methylobacteriaceae*	Unidentified genus
*Moraxellaceae*	*Acinetobacter* (−)
*Neisseriaceae*	*Kingella* (−)
*Propionibacteriaceae*	*Curtobacterium* (+)
	*Propionibacterium* (+)
*Pseudomonadaceae*	*Pseudomonas* (−)
*Ralstoniaceae*	*Ralstonia* (−)
*Rickettsiaceae*	*Rickettsia* (−)
	*Wolbachia* (−)

### Correlation in diversity and abundance

*Lutzomyia intermedia* microbiota diversity and abundance varied based on the studied groups. In the UN and GR groups, the family *Enterobacteriaceae* was the most abundant, representing 54 % and 47 % of each group, respectively. Interestingly, in the BF group, the family *Rickettsiaceae* was the most prevalent, with 46.7 % of the group represented by *Wolbachia* and only 4.3 % of the group represented by *Enterobacteriaceae*. The genera *Ochrobactrum, Bradyrhizobium* and *Pseudomonas* were found across all of the groups.

**Fig. 1:**
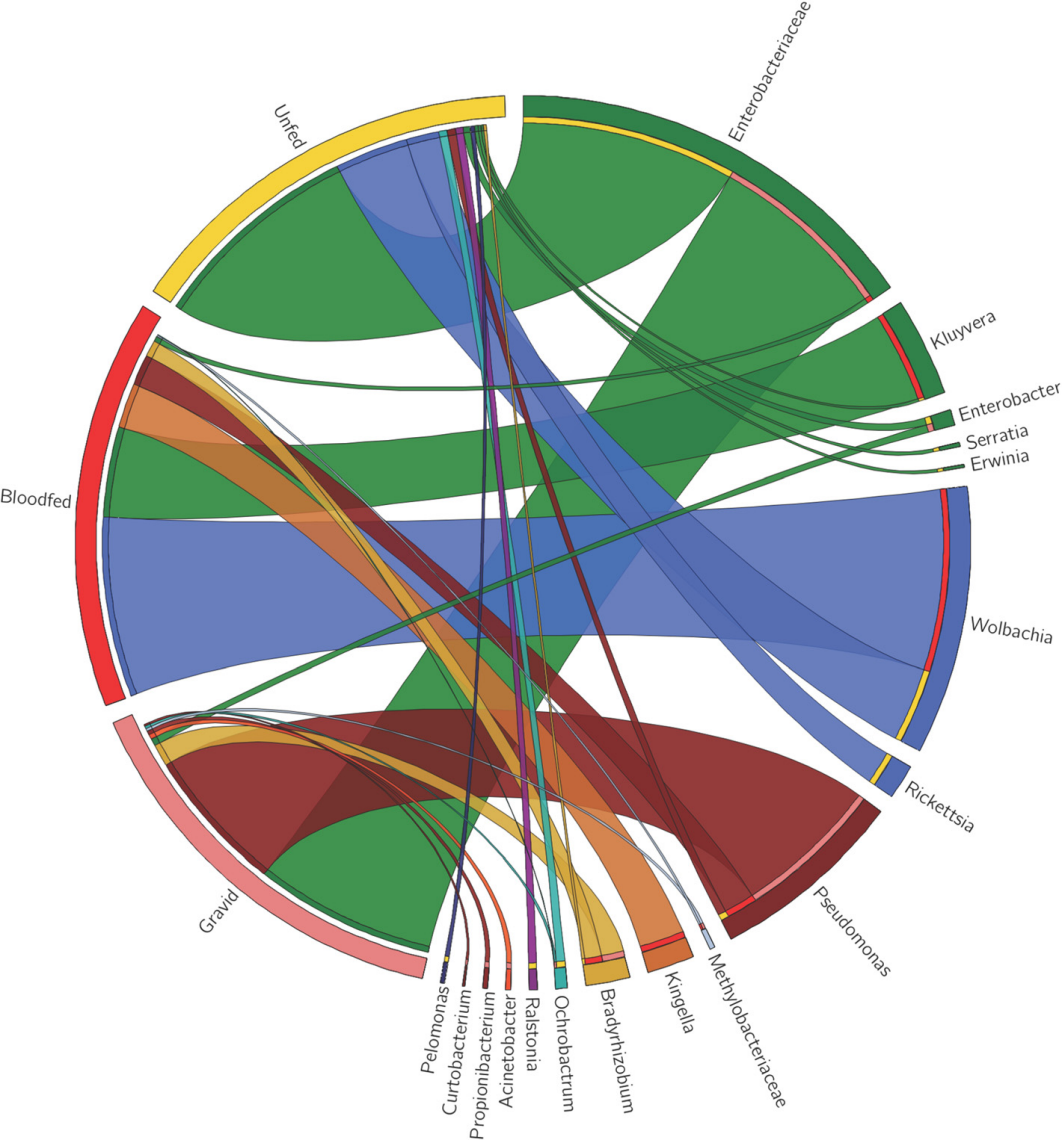
The circular web represents the relative abundance of the bacterial components of *Lutzomyia intermedia* microbiota identified by New Generation Sequencing. Generated with Circos online tool [39]

The abundance of the genera *Bradyrhizobium* and *Pseudomonas* increased after the blood meal (6.0-fold and 3.8-fold, respectively), but abundance increased more in gravid sand flies (8.2-fold and 18.0-fold, respectively). Particularly, the abundance of *Ochrobactrum* bacteria decreased 2.2-fold and 3.7-fold in the BF and GR sand fly groups, respectively (Fig. 1).

## Discussion

Knowledge of the microbiota in the vectors of human-borne diseases is essential to comprehend the relationships with the pathogens they transmit. To our knowledge, this is the first investigation of the native microbiota of *Lu. intermedia*, a proven vector of *L. braziliensis*. Sand flies were collected near Corte de Pedra, a small village and cutaneous leishmaniasis endemic area where 2,384 new cases of ACL were registered in 2015 [27]. Metagenomic analysis revealed only two phyla: (i) Proteobacteria, a dominant phylum with ten families and 13 genera, and (ii) Actinobacteria, with only one family and two genera.

The majority of these families originate from the environment [28–30]. Sand flies can acquire several species of bacteria from the soil during larva nurturing and in plant sap during adult feeding [10–13]. The most abundant family in the UN and GR groups was the *Enterobacteriaceae*, while the *Rickettsiaceae* was the most prevalent in the BF group. The *Enterobacteriaceae* is described as the most common family present in insects. Although the majority of the data was obtained from the UN and GR groups (64,917 and 20,809 reads, respectively), it was not possible to identify the *Enterobacteriaceae* members into genera taxon due to the similarity of the 16S rRNA variable regions [31]. In fact, these data are supported by other studies that detected members of *Enterobacteriaceae* in New and Old World sand flies using cultivation-dependent techniques [9, 32–34].

Three genera were present in *Lu. intermedia* in all experimental groups: *Ochrobactrum, Bradyrhizobium* and *Pseudomonas*. This is the first finding of *Ochrobactrum* in wild New World sand flies, as it was previously reported only in *P. duboscqi*, an Old World leishmaniasis vector and in *Lu. longipalpis* [10, 18]. Similar to our study, *Bradyrhizobium* was described in *Lu. longipalpis* and *Lutzomyia cruzi,* both New World leishmaniasis vectors [35]. *Pseudomonas* was found in both genera, *Phlebotomus* and *Lutzomyia*, from Old and New World sand flies, including *Lu. longipalpis* [11, 31, 32, 36], *P. argentipes*, *P. duboscqi*, *P. papatasi*, *P. perfiliewi* and *P. sargenti* [9, 10, 13, 33, 37, 38]*.*

We assumed that the microbiota from the midgut of unfed *Lu. intermedia* sand flies is a quiescent community of bacteria. These sand flies had no previous contact with blood meal; as such, this group was considered a control group and was used to proportionally compare the abundance of bacterial genera present in the other groups. Fed sand flies showed a 33.4-fold and 2.45-fold increase in the abundance of *Kluyvera* and *Wolbachia*, respectively, compared to controls due to the presence of blood meal in the midgut. Moreover, the abundance of the genera *Bradyrhizobium* and *Pseudomonas* also increased after the blood meal (6.0-fold and 3.8-fold, respectively), but the abundance increased more in gravid sand flies (8.2-fold and 18.0-fold, respectively).

Particularly, the abundance of *Ochrobactrum* bacteria decreased 2.2-fold and 3.7-fold in the blood-fed and gravid sand flies, respectively. Curiously, the *Enterobacter* present in the unfed sand flies were not detected in the blood-fed sand flies but were present at the same abundance in the gravid sand flies. It is interesting to observe that *Kluyvera* and *Pseudomonas*, both found in soil and plants, and *Wolbachia*, a maternally transmitted endosymbiont, are the microbiota genera that were most enhanced by the presence of blood meal in the midgut.

The family *Enterobacteriacea* remained present as the second most abundant after the blood meal represented by the genus *Kluyvera*. In our opinion, this is expected since this family is favored in hematophagous insects after the blood meal. In 2011, Wang et al. [15] showed that members of *Enterobacteriacea* proliferate under oxidative stress due to their capacity to cope with the oxidative stress. Interestingly, *Wolbachia* spp. were most predominant. *Wolbachia* spp. are intracellular organisms and for this reason are protected from the highly oxidative environment in the gut lumen. Until today, the nature of the relation between *Wolbachia* spp. and sand flies is still obscure.

## Conclusion

Our metagenomic analysis revealed that the microbiota of female *Lu. intermedia* sand flies are distinct under specific physiological conditions and consist of 15 bacterial genera with *Ochrobactrum, Bradyrhizobium* and *Pseudomonas* being the common genera. We demonstrated that the presence of a blood meal influences bacterial abundance in the midgut, in a more exacerbated way than the gravid condition. Our results detailing the constituents of *Lu. intermedia* native microbiota contribute to the knowledge of bacterial communities in an important sand fly vector and allow for further studies to better understand how the microbiota interacts with vectors of human parasites and to develop tools for biological control.

## Abbreviations

ACL: American cutaneous leishmaniasis
BF: Blood-fed
GR: Gravid
OTUs: Operational taxonomic units (OTUs)
QIIME: Quantitative insights into microbial ecology
RDP: Ribosomal database project
UN: Unfed
